# Commentary: SLAM- and Nectin-4-Independent Noncytolytic Spread of Canine Distemper Virus in Astrocytes

**DOI:** 10.3389/fmicb.2016.02011

**Published:** 2016-12-09

**Authors:** Giovanni Di Guardo, Roberto Giacominelli-Stuffler, Sandro Mazzariol

**Affiliations:** ^1^Faculty of Veterinary Medicine, University of TeramoTeramo, Italy; ^2^Department of Comparative Biomedicine and Food Hygiene, University of PadovaPadova, Italy

**Keywords:** *Canine Distemper Virus*, *Cetacean morbillivirus*, *Measles Virus*, brain, astrocytes, viral neuropathogenesis, comparative neuropathology

As in the case of the small subset (8–20 per 1 million) of *Measles Virus* (MeV)-infected humans developing a peculiar neurological disease condition known as “subacute sclerosing panencephalitis” (SSPE) (Garg, 2008; Kweder et al., 2015), *Canine Distemper Virus* (CDV) may also give rise to a persistent, “brain-only” form of disease in dogs, known as “old dog encephalitis” (ODE) (Reuter and Schneider-Schaulies, 2010; Sato et al., 2012). Interestingly, peculiar forms of morbilliviral disease resembling those reported in MeV-infected patients and CDV-infected dogs have been also described among *Cetacean Morbillivirus* (CeMV)-infected striped dolphins (*Stenella coeruleoalba*) after the two major morbilliviral epidemics occurred in 1990–92 and 2006–08 in the Western Mediterranean. In a similar manner to what seen in CDV-affected canines and MeV-affected humans, dolphins hit by this form of infection harbor morbilliviral genome and/or antigens exclusively in their brain parenchyma (Domingo et al., 1995; Di Guardo et al., 2013).

Is this enough to conclude that the aforementioned cases of “brain-only,” *Morbillivirus* infection in striped dolphins could/should be regarded as reliable, comparative neuropathology and viral neuropathogenesis models in relation to their canine and human “counterparts”?

We don't know, although “yes and no” seems to be the best possible answer at the moment, provided that the agent- and host-related factors and mechanisms driving CeMV colonization and persistence inside the brain of chronically infected dolphins are unknown (Di Guardo et al., 2013; Di Guardo and Mazzariol, 2016). In this respect, *Signaling Lymphocyte Activation Molecule* (SLAM/CD150), the cell receptor involved in the well-documented lymphotropic behavior of *Morbillivirus* genus members, is not expressed by neurons, similarly to nectin-4, another receptor molecule accounting for morbilliviral epitheliotropism (Sato et al., 2012). Nevertheless, it has also been suggested that nectin-4 expression could be related to CDV neurovirulence, with nectin-4-immunoreactive neurons of the canine brain representing a preferential virus target (Pratakpiriya et al., 2012). Worthy to be mentioned, the long-lasting persistence of CDV in the brain tissue from ODE-affected dogs has been recently described as the result of a non-cytolytic, astrocyte-to-astrocyte viral spread through a putative, hitherto unknown glial cell receptor, different from SLAM and nectin-4, which has been provisionally termed “GliaR” (Alves et al., 2015). This is of special concern in relation to “canine demyelinating leukoencephalitis,” one of the various disease conditions suffered by CDV-infected dogs, a peculiar feature of which is represented by the viral colonization of vimentin-positive astrocytes, a population of immature and/or reactive astroglial cells that might support CDV persistence and spread within the brain of chronically infected dogs (Lempp et al., 2014), thus allowing the virus to “escape” the host's intrathecal immune response (Alves et al., 2015). Anyway, it should be additionally underscored that, despite the well-documented propensity of CDV to colonize the host's central nervous system (CNS), there are remarkable differences among the various CDV strains in terms of neurovirulence, neurological disease phenotypes and CNS areas targeted by the virus, with the molecular determinants specifically accounting for such differences being largely unknown. For instance, the Snyder Hill CDV strain, differently from the aforementioned “canine demyelinating leukoencephalitis” model (Lempp et al., 2014; Alves et al., 2015), is highly neurovirulent, being responsible for a rapidly progressive and lethal polioencephalitis (Summers et al., 1984).

Notwithstanding the above, a very limited viral colonization of astrocytes has been recently observed by us in the cerebral tissue from three striped dolphins stranded along the Italian coastline, all carrying a “brain-only” form of CeMV infection. Still of interest, a quite prominent astrogliosis and astrocytosis have been simultaneously detected in the brains from these dolphins (Di Guardo et al., in press).

The brain pathomorphological changes' pattern(s) should not be viewed, however, as the only “parameter” upon which building a comparison between human SSPE, canine ODE and the “brain-only” forms of morbilliviral disease that are being increasingly reported among Mediterranean striped dolphins (as well as in dolphins from other geographical areas of our Planet) (Di Guardo and Mazzariol, 2016). As a matter of fact, the “wild type” MeV strains/genotypes causing human SSPE are known to harbor a number of mutations, more frequently involving the viral matrix protein (M) gene and, to a lesser extent, also the fusion protein (F) and the haemagglutinin (H) genes. Furthermore, an enhanced viral spread, associated with a compromised capability of MeV strains/genotypes to produce infectious, cell-free viral particles, have been related to the aforementioned gene mutations (Garg, 2008; Moulin et al., 2011; Kweder et al., 2015).

To our knowledge, studies of this kind have never been performed on striped dolphins affected by “brain-only” forms of CeMV infection. The same is true as far as nectin-4 expression within the cetacean CNS is specifically concerned. Thanks to similar investigations, indeed, it could be assessed “whether” and to “which extent,” among others, striped dolphins with “brain-only” forms of CeMV infection may be regarded as “models” for the comparative neuropathology and viral neuropathogenesis study of human SSPE in MeV-infected patients, as well as of ODE in CDV-infected dogs.

## Author contributions

GDG wrote the first draft of the Commentary, that was critically revised by RGS and SM. The original manuscript's text/version was entirely agreed by the three authors. The same also applies to the revised manuscript's text.

### Conflict of interest statement

The authors declare that the research was conducted in the absence of any commercial or financial relationships that could be construed as a potential conflict of interest.
